# Diagnostic reference levels in interventional radiology: a systematic review

**DOI:** 10.1590/0100-3984.2025.0016

**Published:** 2025-10-21

**Authors:** Iana Quintanilha de Borba, Rochelle Lykawka, Nayron Medeiros Soares, Joaquim Maurício da Motta Leal Filho, Alexandre Bacelar, Matheus de Lima Ruffini, Adolfo Moraes de Souza, Fabiano Reis, Juliana Ávila Duarte

**Affiliations:** 1 Universidade Federal do Rio Grande do Sul (UFRGS), Porto Alegre, RS, Brazil.; 2 Hospital de Clínicas de Porto Alegre (HCPA), Porto Alegre, RS, Brazil; 3 Universidade Federal de Ciências da Saúde de Porto Alegre (UFCSPA), Porto Alegre, RS, Brazil; 4 Instituto do Coração do Hospital das Clínicas da Faculdade de Medicina da Universidade de São Paulo (InCor/HC-FMUSP), São Paulo, SP, Brazil; 5 Instituto do Câncer do Estado de São Paulo Octavio Frias de Oliveira (Icesp), São Paulo, SP, Brazil; 6 Faculdade de Ciências Médicas da Universidade Estadual de Campinas (FCM-Unicamp), Campinas, SP, Brazil

**Keywords:** Diagnostic reference levels, Radiology, interventional, Radiation protection, Fluoroscopy, Níveis de referência de diagnóstico, Radiologia intervencionista, Proteção radiológica, Fluoroscopia

## Abstract

**Objective:**

To comprehensively and impartially analyze the scientific evidence available for establishing diagnostic reference levels (DRLs) in interventional radiology.

**Method:**

This was a systematic review conducted in accordance with the Preferred Reporting Items for Systematic Reviews and Meta-Analyses guidelines. The search focused on studies related to interventional radiology and DRLs in PubMed/Medline and Embase. Studies involving computed tomography-guided procedures, studies with incomplete data, and systematic reviews were excluded. Two independent reviewers evaluated the studies, resolving discrepancies with a third reviewer. Articles were tabulated with information such as title, publication year, procedures, DRL values, and type of equipment used.

**Results:**

A total of 475 articles were identified. After duplicates had been excluded and eligibility criteria had been applied, the final sample comprised 30 articles. Most DRL values (73%) were reported at the local level, as defined by International Commission on Radiological Protection criteria, representing typical dose values from a sample within one or a few institutions. A total of 113 procedures were identified, with endovascular aneurysm repair and nephrostomy being the most frequently reported. We identified DRLs at national and regional scales, predominantly within Europe. Influencing factors included technology, operator experience, specific protocols, and optimization strategies. The analysis also identified a lack of longitudinal studies assessing changes over time. The use of dose management software emerged as an effective tool for facilitating data collection and DRL establishment.

**Conclusion:**

The lack of standardized procedural terminology hindered direct DRL comparisons. Our findings highlight a predominance of European studies and emphasize the need for broader international efforts to improve DRL implementation.

## Abstract (pt)

**Objetivo:**

Analisar de forma abrangente e imparcial as evidências científicas disponíveis na literatura para o estabelecimento de níveis de referência diagnósticos (DRLs) em radiologia intervencionista.

**Método:**

Foi realizada uma revisão sistemática seguindo as diretrizes da declaração Preferred Reporting Items for Systematic Reviews and Meta-Analyses. A busca concentrou-se em estudos relacionados à radiologia intervencionista e aos DRLs, utilizando estratégias de busca nas bases PubMed/Medline e Embase. Foram excluídos estudos envolvendo tomografia computadorizada, estudos incompletos e revisões sistemáticas. As avaliações foram conduzidas de forma independente por dois revisores, com resolução de discrepâncias por um terceiro avaliador. Os artigos selecionados foram tabulados, incluindo informações como título, ano de publicação, procedimentos e valores de DRLs.

**Resultados:**

Um total de 475 artigos foi identificado. Após a exclusão de duplicatas e a aplicação dos critérios de elegibilidade, 30 artigos foram incluídos na análise. A maioria dos valores de DRL (73%) foi relatada em nível local, conforme definido pelos critérios da International Commission on Radiological Protection, representando valores de dose típicos de uma amostra dentro de uma ou algumas instituições. Foram identificadas 113 diferentes denominações de procedimentos, sendo o reparo endovascular de aneurisma e a nefrostomia os mais frequentes. Valores de DRL em níveis nacional e regional também foram identificados, com predominância europeia. A análise destacou a influência de fatores como tecnologia, experiência do operador e protocolos específicos, além de estratégias de otimização. A análise de tendências revelou a carência de estudos longitudinais avaliando mudanças ao longo do tempo. O uso de *softwares* de gerenciamento de dose mostrou-se uma ferramenta eficaz para a coleta de dados e o estabelecimento de DRL.

**Conclusão:**

A falta de padronização nas denominações dos procedimentos representou um desafio para a comparação direta dos valores de DRL entre os estudos. Os achados também ressaltaram a escassez de estudos em regiões fora da Europa.

## INTRODUCTION

Interventional radiology is a constantly evolving field, playing an essential role in the diagnosis and treatment of a wide variety of clinical conditions^(^1^)^. Radiation exposure remains a significant concern, particularly in fluoroscopy-guided interventional (FGI) procedures. Although the clinical benefits usually outweigh the risks associated with X-ray exposure, minimizing exposure whenever possible is crucial^(^2^)^. This is especially important for high-dose procedures, which require continuous monitoring and optimization.

The concept of diagnostic reference levels (DRLs) was introduced by the International Commission on Radiological Protection (ICRP) in Publication 73^(^3^)^. In ICRP Publication 135^(^4^)^, DRLs were established as an optimization strategy, serving as quality indicators for procedural performance^(^3^,^5^)^. Rather than being patient dose limits, DRLs are reference values statistically determined for standard patients to guide periodic institutional dose evaluations aimed at adhering to the “as low as reasonably achievable” principle^(^3^)^. Although DRLs constitute a valuable tool for optimizing patient radiological protection, challenges remain regarding the methodology for establishing and applying these values^(^6^,^7^)^. Those challenges are particularly pronounced for interventional diagnostic or therapeutic procedures, for which procedural complexity varies significantly.

The considerable variation in radiation doses across interventional radiology procedures suggests a need for greater attention to the variables influencing DRL values, with the objective of optimizing patient safety^(^7^)^. The aim of this study was to conduct a comprehensive, impartial systematic review of the scientific evidence for establishing DRLs in interventional radiology through an analysis of observational studies.

## METHOD

This systematic review followed the Preferred Reporting Items for Systematic Reviews and Meta-Analyses guidelines^(^8^,^9^)^. Studies published up through August 2023 were considered. A filter was applied to select studies published from 2017 onward, covering a six-year period. Searches were conducted in the PubMed/Medline and Embase databases. Although a search was attempted in the Cochrane Library, no relevant results were found.

The search strategy included descriptors and their variations: *Diagnostic reference levels*; *Radiology, interventional*; *Fluoroscopy*; *Tomography, X-Ray computed*; *Cholangiography*; *Image-guided biopsy*; *Catheterization*; *Balloon angioplasty*; *Cineradiography*; *Photofluorography*; *Cholangiography*; *Percutaneous transthoracic biopsy*; *Artery embolization*; *Neuroradiography*; *Cerebral ventriculography*; *Subtraction technique*; *Angiography*; *Cineangiography*; *Phlebography*; *Portography*; *Coronary angiography*; and *Arthrography*.

The following inclusion criteria were applied: studies reporting DRL values for air kerma-area product (KAP), cumulative air kerma (CAK), and fluoroscopy time (FT); studies on FGI radiology procedures in adults and children; and studies using DRL metrics according to ICRP Publication 135 (median or 75th percentile). Studies on computed tomography-guided interventional radiology procedures were excluded, as were studies with incomplete data and systematic reviews. The protocol was registered in the International Prospective Register of Systematic Reviews under registration number CRD42023446225.

The articles were exported from the PubMed/Medline and Embase databases to an artificial intelligence-powered tool for systematic literature reviews (Rayyan; Qatar Computing Research Institute, Doha, Qatar). Initially, duplicate articles were automatically removed with Rayyan. Thereafter, two reviewers independently conducted the initial assessment phase, involving the analysis of abstracts and, finally, full-text articles. This process resulted in a collection of studies for evaluation by the assessors. A third assessor resolved discrepancies in selection to reach a consensus. In the consensus meeting, articles not aligned with the objectives of this review were excluded.

After articles had been selected for full-text review, they were exported and tabulated by one of the reviewers. The extracted data included the article title; digital object identifier; year of publication; procedures described; DRL values (KAP, CAK, and FT); and the type of equipment used. The identification of 113 procedures was based on the nomenclature as reported in the included studies, without reclassification or grouping into broader procedural categories. In addition, we determined whether the studies considered factors such as procedure complexity, patient age, patient body habitus, study limitations, practical recommendations, trend analysis, potential research gaps, and variability factors.

## RESULTS

### Study sample

**Figure 1:**
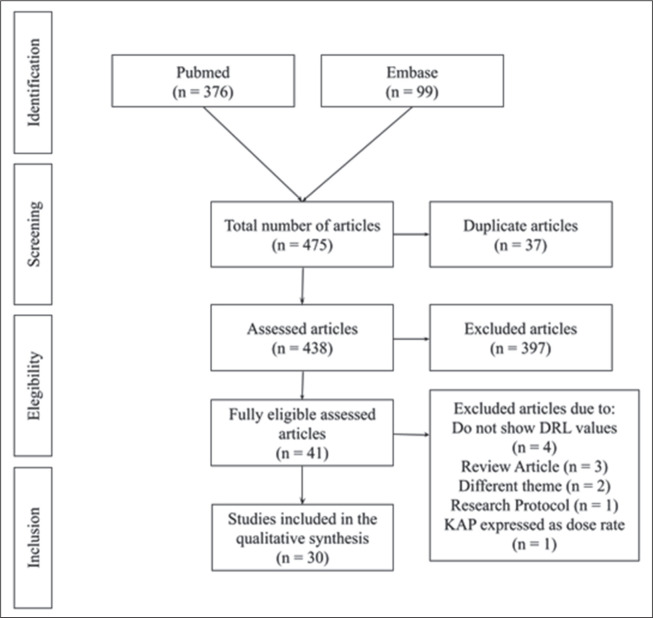
Flow chart of the article selection process, after searches in the PubMed and Embase databases.

In the initial screening, 376 articles were found in PubMed and 99 in Embase, totaling 475 articles. Of those, 37 articles were excluded for being in more than one database (duplicates) and 397 did not meet the eligibility criteria, leaving 41 articles eligible for evaluation. The final selection process resulted in the inclusion of 30 articles. All articles assessed DRL in various FGI procedures. The selection process is depicted in Figure 1.

In total, 113 different types of FGI procedures were identified, with the most common being endovascular aneurysm repair (EVAR) procedures (n = 4) and nephrostomy (n = 3). Procedures were identified based on the descriptions provided in the studies, which primarily referenced the technical procedure performed rather than the clinical indication, equipment used, or detailed patient characteristics. Few procedures were repeated across studies, making it challenging to compare the DRL values obtained.

Only four studies^(^10^–^13^)^ addressed local DRLs for pediatric procedures, the remainder involving only adult patients. In the studies dedicated to pediatric procedures, the analysis of results was stratified based on the weight/height or age of the patients. In contrast, in the studies involving adult patients, the evaluation was performed for patients established as the standard.

### Geographic distribution

According to ICRP Publication 135, a local DRL refers to the typical dose values derived from a sample within a single institution or a small group of facilities, representing standard clinical practice at the local level. Of the 30 studies analyzed, 23 (76.7%) reported dose values referenced as local/institutional DRLs, five (16.7%) reported national DRLs, and two (6.7%) reported regional DRLs. One study^(^14^)^, conducted in Malta, provided local DRLs for eight procedures, which also represented the national DRLs, because the study was conducted at the sole institution performing those procedures in the country. Another study^(^15^)^ presented the local DRL for a procedure that also represented the regional (European) DRL, because it brought together the major centers performing the EVAR procedure in the region.

**Figure 2:**
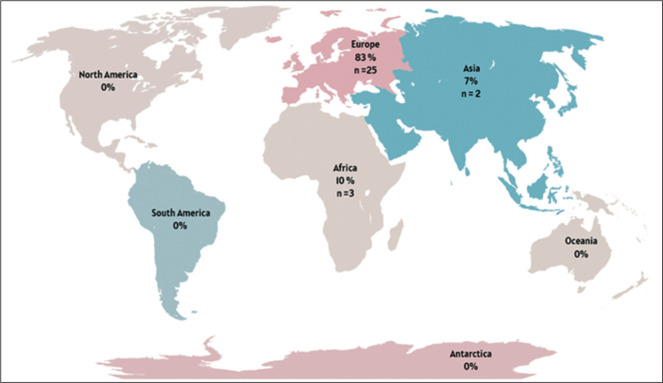
Number/percentage of studies by region.

The geographic distribution of the studies included is shown in Figure 2. The European continent showed the highest number of DRL studies in interventional radiology, with the majority of those studies (n = 13) conducted in Germany. None of the studies selected were conducted in North America, Latin America, the Caribbean, Oceania, or Antarctica.

### Reported dose descriptors

Although all of the studies provided a DRL for KAP, only 13 (43.3%) included the DRL for CAK value, whereas 16 (53.3%) included the DRL for FT. Only nine studies (30.0%) presented DRL values for all three dose descriptors (KAP, CAK, and FT). Some studies reported the mean FT, which was not taken into account in the present study.

### DRL value

National DRLs were identified for Malta, Spain, France, Lebanon, and Germany. Table 1 presents those DRL values. National DRL information was collected through survey forms sent to eligible institutions. However, researchers encountered challenges in conducting these studies, including a low response rate to the questionnaires^(^16^)^, a lack of dose correction factors in KAP data^(^17^)^, and potential typing errors in manual data collection^(^14^)^, as well as a lack of CAK and exposure time data. In addition, all studies reported difficulty in assessing the complexity of procedures was reported in all of the studies. Pace et al.^(^14^)^ recommended the use of dose management software to facilitate data collection.

**Table 1:** National DRL values described in the articles evaluated.

Country	Study	Procedure	Sample (n)	DRL value*		
				KAP (Gycm^2^)	CAK (Gy)	FT (min)
Malta	Pace et al.^(^14^)^	Central lines	269	1	–	–
		Embolization	297	58	–	–
		Hepatic embolization	215	96	–	–
		Mechanical thrombectomy	122	120	–	–
		Nephrostomy single	148	2	–	–
		PICC lines	135	0.3	–	–
		PTA	762	5	–	–
		PTC	238	8	–	–
Spain	Rial et al.^(^18^)^	EVAR - mobile X-ray systems	165	87	0.292	–
		EVAR - hybrid rooms	123	278	1.403	–
France	Farah et al.^(^16^)^	Abdominal aortic aneurysm endoprosthesis	–	81	–	18
		Iliac angioplasty	–	24	–	6
		Flutter ablation	–	14	–	17
Lebanon	Rizk et al.^(^17^)^	Cerebral embolization	117	190	2.42	27
		Cerebral arteriography	210	83	0.69	6
		Lower extremity arteriography	343	31	0.17	3
		Lower extremity arteriography with coronary angiography	86	43	0.41	5
		Lower extremity angioplasty	177	48	0.27	12
		Inferior vena cava filter	26	57	0.31	7
Germany	Schmitz et al.^(^19^)^	Initial percutaneous biliary interventions	240	43	–	–
		Follow-up percutaneous biliary interventions	320	14	–	–

* Reported as the third quartile. PICC, peripherally inserted central catheter; PTA, percutaneous transluminal angioplasty; PTC, percutaneous transhepatic cholangiography.

Local DRLs from small countries like Malta^(^14^)^ were treated as national DRLs. The selected studies encompassed various public and private institutions in different countries, including 14 hospitals in Spain, 150 in France, 15 in Lebanon, and 23 in Germany. It is important to note that none of the studies used previously published or compiled data, opting for retrospective and prospective data collection after including institutions. In the context of national DRL studies, the highest recorded value was for the EVAR procedure in hybrid rooms, with DRLs for the KAP and CAK of 278 Gycm^2^ and 1,403 Gy, respectively.

The two studies reporting regional DRLs were from Europe and described distinct procedures. Regional DRLs were defined in centers from various countries. One of the studies highlighting the EVAR procedure^(^15^)^ included large and medium-sized hospitals in Ireland and Italy, whereas the other^(^19^)^ included 16 hospitals in 13 different European countries. Table 2 compiles the data found for regional DRL values. Schegerer et al.^(^20^)^ reported that, despite selecting the largest centers in European countries over a 12-month period, they were not able to acquire a sample of 20 patients for eligible procedures at some centers. Similar to what was found for national DRLs, the authors of both studies reported that complexity was not considered and suggested that this analysis be included in future research. Within regional DRL studies, the highest values were found for hepatic embolization (transarterial chemoembolization), with DRLs for KAP and CAK of 241 Gycm^2^ and 1.868 Gy, respectively. In addition, 19 studies exclusively dedicated to local DRLs were identified, established in a sample of centers within a country, as outlined in Table 3, along with the two previously mentioned studies highlighting the interconnection of local DRLs across regional and national categories^(^14^,^15^)^.

**Table 2:** Regional DRL values described in the articles evaluated.

Region	Study	Procedure	Sample (n)	DRL value *		
				KAP (Gycm^2^)	CAK (Gy)	FT (min)
Europe	Schegerer et al.^(^20^)^	Iliac artery stenting	–	58	0.251	10
		Hepatic embolization (transarterial chemoembolization)	–	241	1.868	18
		Femoropopliteal artery stenting	–	26	0.99	13
		Treatment of biliary obstruction	–	23	1.95	10
Europe	Tuthill et al.^(^15^)^	Abdominal EVAR	180	158.49	–	18.13

* Reported as the third quartile.

**Table 3:** Local DRL values described in the articles evaluated.

Country	Study	Procedure	Sample (n)	DRL value*		
				KAP (Gycm^2^)	CAK (Gy)	FT (min)
Korea	Ihn et al.^(^21^)^	Diagnostic cerebral angiography	429	101.6	0.711	13.3
		Aneurysm coiling	327	199.9	3.458	57.3
		Stroke thrombolysis	326	225.1	1.590	44.7
		Arteriovenous malformation embolization	78	412.3	4.447	99.3
Italy	Isoardi et al.^(^22^)^	Cerebral angiography	981	159	1.401	10
Germany	Forbrig et al.^(^23^)^	Endovascular carotid artery stenting	102	117	–	–
		Semi elective/elective carotid artery stenting	75	86.7	–	27.1
		Carotid artery stenting + mechanical thrombectomy	19	286.1	–	43.8
Greece	Vossou et al.^(^24^)^	Hepatic chemoembolization	218	141	0.634	12.4
		Iliac stent placement		130	0.330	17.9
		Femoropopliteal revascularization		28	0.112	–
Germany	Opitz et al.^(^25^)^	Endovascular treatment of UIAs	583	183	–	–
		Endovascular treatment of ruptured intracranial aneurysms		246	–	–
Germany	Ozpeynirci et al.^(^26^)^	Endovascular coil embolization in carotid-cavernous fistulas	30	376.2	–	241.8
Germany	Ozpeynirci et al.^(^27^)^	Spinal angiography in spinal dural arteriovenous fistulas	62	329.41	–	–
Germany	Opitz et al.^(^28^)^	Diagnostic angiography in carotid-cavernous fistula	26	215	–	–
		Embolization in carotid-cavernous fistula	60	350	–	–
Germany	Opitz et al.^(^29^)^	Endovascular therapy in cranial dural arteriovenous fistula	94	507.33	–	–
		Diagnostic angiography in cranial dural arteriovenous fistula		256.65	–	–
		Endovascular therapy in spinal dural arteriovenous fistula	37	482.72	–	–
		Diagnostic angiography in spinal dural arteriovenous fistula		396.39	–	–
Greece	Papanastasiou et al.^(^30^)^	Cerebral angiography	142	70	0.494	9.2
		PTC		34	0.194	14.2
		Transarterial chemoembolization		189	1.186	27.5
		Percutaneous transhepatic biliary drainage		54	0.400	22.9
Germany	Forbrig et al.^(^31^)^	Endovascular treatment in patients with intracranial lateral dural arteriovenous fistulae	70	414	–	142
South Africa	Peter et al.^(^32^)^	Intracranial aneurysm coil embolization	30	52.1	–	17.8
South Africa	Malan et al.^(^33^)^	Leg – aorto-bifemoral angiogram	590	51	0.15	6
		Leg – lower limb angiogram (trauma)	70	42	0.16	8
		Diagnostic cerebral angiogram	61	55	0.289	14
		Leg – aorto-bifemoral intervention	287	73	0.249	18
		Nephrostomy (unilateral)	265	10	0.063	4
		Bronchial artery embolization	208	73	0.259	38
		PTC drainage	173	46	0.227	20
		Nephrostomy (bilateral)	147	9	0.057	4
		Nephrostomy and stent (unilateral)	122	39	0.196	12
		Selective abdominal vessels – interventional angiogram	77	170	0.877	29
		Selective upper extremity – interventional angiogram (trauma)	73	30	0.094	13
		Selective neck vessel – interventional angiogram (trauma)	65	75	0.587	27
		Interventional cerebral angiogram	55	63	0.505	25
		PTC stent ± dilatation	54	80	0.443	19
		Nephrostomy and stent (bilateral)	45	54	0.342	24
Greece	Tzanis et al.^(^34^)^	EVAR ≥ 5	24	196.2	1.239	23.8
		EVAR 5–7	35	244.6	1.358	31.1
		EVAR > 7	14	375.6	2.284	44.1
		EVAR	76	230.6	–	–
Germany	Forbrig et al.^(^35^)^	Endovascular treatment of UIAs – coiling	26	130	–	–
		UIAs – flow diverter and Woven EndoBridge	45	176	–	–
		UIAs – combined techniques	16	209	–	–
Ireland	Acton et al.^(^36^)^	Four-vessel angiogram	189	96	–	–
		Aneurysm coiling	109	123	–	–
Greece	Metaxas et al.^(^37^)^	Cervical interventions	45	0.10	0.0047	0.15
		Thoracolumbar interventions	111	0.71	0.0032	0.29
South Africa	Slave et al.^(^38^)^	Percutaneous transhepatic biliary drainage	146	24	131.8	6.2
		Bronchial artery embolization	57	131	343	33.5
		Pigtail insertion	44	7.5	37	2.4
		Nephrostomy (unilateral)	42	10	26	3.4
		Nephrostomy (bilateral)	37	10	62	6.3
		Selective abdominal vessels-interventional angiogram	26	776	2227.8	28.3
		Diagnostic cerebral angiogram	26	209.3	868.5	28.4
		PICC	25	2	5	4
		Percutaneous transhepatic biliary drainage internalization	20	57	259	16.7
		Uterine artery embolization	18	1463.8	4019	24.8
		Unilateral antegrade ureteric stent	16	23	118.5	15.2
		PTC	15	9	28.5	0.7
		Interventional cerebral angiogram	15	275	1744	34.1
Switzerland	Heilmaier et al.^(^39^)^	Insertion of abscess drainage – simple	10	2	–	–
		Insertion of abscess drainage – standard	10	6	–	–
		Insertion of abscess drainage – difficult	6	13	–	–
Switzerland	Heilmaier et al.^(^39^)^	Nephrostomy insertion – simple	12	5	–	–
		Nephrostomy insertion – standard	29	12	–	–
		Nephrostomy insertion – difficult	13	30	–	–
		Nephrostomy change/removal – simple	58	6	–	–
		Nephrostomy change/removal – standard	31	8	–	–
		Nephrostomy change/removal – difficult	8	25	–	–
		Percutaneous radiologic gastrostomy tube insertion – standard	6	9	–	–
		Percutaneous radiologic gastrostomy control – standard	7	8	–	–
		PTC drain insertion – simple	7	32	–	–
		PTC drain insertion – standard	41	40	–	–
		PTC drain insertion – difficult	20	85	–	–
		PTC drain change/removal – simple	21	12	–	–
		PTC drain change/removal – standard	29	20	–	–
		PTC drain change/removal – difficult	11	60	–	–
		Selective internal radiotherapy – standard	18	175	–	–
		Transarterial chemoembolization – standard	11	210	–	–
		Transarterial chemoembolization – difficult	7	310	–	–
		Transjugular liver biopsy – simple	6	18	–	–
		Transjugular liver biopsy – standard	9	35	–	–
		Transjugular liver biopsy – difficult	6	50	–	–
		Intervention to superior vena cava – standard venous	14	10	–	–
		Intervention to inferior vena cava – simple	11	22	–	–
		Intervention to inferior vena cava – standard	6	35	–	–
		Fluoroscopy of port-a-cath – simple	9	2	–	–
		Fluoroscopy of port-a-cath – standard	7	5	–	–
		Insertion of PICC – simple	92	1	–	–
		Insertion of PICC – standard	56	2	–	–
		Insertion of PICC – difficult	11	22	–	–
		Embolization therapy of varicocele – simple	10	25	–	–
		Embolization therapy of varicocele – standard	7	40	–	–
		Embolization therapy of varicocele – difficult	6	55	–	–
		Phlebography, lower extremity – standard	6	2	–	–
		Insertion of dialysis graft – simple arterial-venous	7	6	–	–
		Insertion of dialysis graft – difficult	5	10	–	–
		Thrombolysis of dialysis graft – standard	15	3	–	–
		PTA of dialysis graft – standard	7	40	–	–
		EVAR – standard	17	185	–	–
		EVAR – difficult	16	350	–	–
		Visceral artery angiography – standard	12	140	–	–
		Visceral artery angiography – difficult	8	245	–	–
		Visceral artery embolization – standard	15	165	–	–
		Visceral artery embolization – difficult	20	430	–	–
		Renal PTA – difficult	6	220	–	–
		Renal artery embolization – standard	6	105	–	–
		Renal artery embolization – difficult	6	195	–	–
		Pelvic vessel embolization (venous/arterial) – standard	11	150	–	–
		Pelvic vessel embolization (venous/arterial) – difficult	12	205	–	–
		Pelvic PTA – simple	7	50	–	–
		Pelvic PTA – standard	26	60	–	–
		Pelvic PTA – difficult	13	95	–	–
		Pelvic PTA and stent placement – simple	11	20	–	–
		Pelvic PTA and stent placement – standard	22	85	–	–
Switzerland	Heilmaier et al.^(^39^)^	Pelvic PTA and stent placement – difficult	28	145	–	–
		Pelvic and femoral PTA – standard	18	65	–	–
		Pelvic and femoral PTA – difficult	16	70	–	–
		Pelvic and femoral PTA and stent placement – standard	13	85	–	–
		Pelvic and femoral PTA and stent placement – difficult	10	100	–	–
		Pelvic, femoral and lower leg PTA – standard	8	25	–	–
		Pelvic, femoral and lower leg PTA – difficult	9	75	–	–
		Diagnostic angiography lower extremity – simple	15	3	–	–
		Diagnostic angiography upper extremity – standard	10	5	–	–
		Insertion of thrombolysis catheter – simple	7	20	–	–
		Insertion of thrombolysis catheter – standard	11	25	–	–
		Insertion of thrombolysis catheter – difficult	8	50	–	–
		Control of thrombolysis catheter – simple	14	4	–	–
		Control of thrombolysis catheter – standard	8	6	–	–
		Femoral PTA – simple	14	8	–	–
		Femoral PTA – standard	59	20	–	–
		Femoral PTA – difficult	14	55	–	–
		Femoral PTA and stent placement – simple	9	6	–	–
		Femoral PTA and stent placement – standard	35	15	–	–
		Femoral PTA and stent placement – difficult	27	45	–	–
		Femoral and lower leg PTA – simple	8	5	–	–
		Femoral and lower leg PTA – standard	49	10	–	–
		Femoral and lower leg PTA – difficult	18	30	–	–
		Femoral and lower leg PTA and stent placement – simple	8	4	–	–
		Femoral and lower leg PTA and stent placement – standard	17	8	–	–
		Femoral and lower leg PTA and stent placement – difficult	20	20	–	–
		Lower leg PTA – simple	10	3	–	–
		Lower leg PTA – standard	42	6	–	–
		Lower leg PTA – difficult	22	25	–	–
		Lower leg PTA and stent placement – standard	15	8	–	–
		Lower leg PTA and stent placement – difficult	15	30	–	–

* Reported as the third quartile. PTA, percutaneous transluminal angioplasty.

Among the 19 studies that defined local DRLs, only two were multicenter studies. The study conducted by Ihn et al.^(^21^)^ involved 22 hospitals, totaling 22 X-ray systems, whereas that conducted by Isoardi et al.^(^22^)^ included 21 hospitals, with a total of 44 hybrid rooms and 16 mobile X-ray units. Slave et al.^(^38^)^ conducted a single-center study with two X-ray systems, and the remaining 16 studies were single-center studies with only one X-ray system^(^23^,^25^–^29^,^31^–^37^,^39^)^. Two studies do not provide details on the institution and X-ray systems used^(^24^,^30^)^. All of the studies were retrospective, except for that conducted by Tzanis et al.^(^34^)^, who described their study design as prospective. It is also noteworthy that the highest local DRLs were associated with uterine artery embolization, with specific values for KAP, CAK, and FT of 1463.8 Gycm^2^, 4.019 Gy, and 24.8 min, respectively.

### Complexity assessment

Only Heilmaier et al.^(^39^)^ and Tzanis et al.^(^34^)^ took into account the level of complexity of the procedures. In the first article, 40 FGI procedures were analyzed, classified as “simple”, “standard”, or “difficult”. To define those levels of complexity, interventional radiologists considered patient cooperation, patient body mass index, standard anatomy, access/puncture difficulties, and complications. In the Tzanis et al. study^(^34^)^, 70 EVAR procedures were included. The authors classified complexity by using a scoring system, considering access vessels (normal, unilateral, or bilateral), aortic neck anatomy, concomitant procedures, and contralateral limb catheterization time. Thus, they distributed the procedures into three categories of complexity: low (total score ≤ 5), medium (total score of 6 or 7), and high (total score > 7).

### Pediatric DRLs

Among the studies selected, all pediatric DRLs were local (Table 4). The authors stratified their samples by body weight or age group. The procedure with the highest pediatric DRL values was sclerotherapy for patients weighing 50–80 kg, with DRL values for KAP and FT of 37.34 Gycm^2^ and 23.3 min, respectively^(^10^)^. Not all authors collected CAK and exposure time data.

**Table 4:** Pediatric DRL values in the articles evaluated.

Country	Study	Procedure	Sample (n)	DRL value*		
				KAP (Gycm^2^)	CAK (Gy)	FT (min)
Spain	Morcillo et al.^(^10^)^	Hepatic/biliary interventions (5–15 kg)	39	13.04	–	19.38
		Hepatic/biliary interventions (15–30 kg)	15	21.21	–	22.65
		Sclerotherapy (15–30 kg)	18	7.04	–	5.9
		Sclerotherapy (30–50 kg)	21	40.49	–	7.45
		Sclerotherapy (50–80 kg)	16	37.34	–	23.3
		Central venous catheters (5–15 kg)	21	0.84	–	3.4
Italy	Gerasia et al.^(^11^)^	Retrograde wedge portography – children	25	5.6	0.034	–
		Retrograde wedge portography – middle childhood	20	6.4	0.018	–
		Retrograde wedge portography – early adolescence	21	12.8	0.059	–
Germany	Opitz et al.^(^12^)^	IAC procedures of pediatric patients with RB – A2: 4–12 months	85	3.9	–	–
		IAC procedures of pediatric patients with RB – A3: 13–72 months	157	7.0	–	–
		IAC procedures of pediatric patients with RB – A4: 73 months-10 years	4	14.5	–	–
France	Farah et al.^(^13^)^	PTC 0–5 kg	7	0.06	0.001	–
		PTC 5–15 kg	56	0.22	0.006	–
		PTC 15–30 kg	43	0.68	0.035	–
		PTC 30–50 kg	42	1.07	0.027	–

* Reported as the third quartile. IAC, intra-arterial chemotherapy; RB, retinoblastoma; PTC, percutaneous transhepatic cholangiography.

### EVAR comparative analysis

The only procedure identified in studies of local, national, and regional DRL values was EVAR, although the EVAR classifications differed among them. As can be seen in the Table 5, the local DRL value for the KAP dose descriptor in the Tzanis et al. study^(^34^)^, which considered a single facility, was higher than the regional DRL value reported for Europe and the national DRL value reported for mobile X-ray systems in Spain (230.6 Gycm^2^ vs. 158.49 Gycm^2^ and 87 Gycm^2^, respectively). However, those values were all lower than the 278.0 Gycm^2^ reported for hybrid rooms in Spain^(^18^)^.

**Table 5:** Comparative regional, national, and local DRL values described for the EVAR procedure in the articles evaluated.

DRL type	Study	Procedure	Sample (n)	DRL value*		
				KAP (Gycm^2^)	CAK (Gy)	FT (min)
Local (Facility in Greece)	Tzanis et al.^(^34^)^	EVAR ≥ 5	24	196.2	1.239	23.8
		EVAR 5–7	35	244.6	1.358	31.1
		EVAR > 7	14	375.6	2.284	44.1
		EVAR	76	230.6	–	–
National (Spain)	Rial et al.^(^18^)^	EVAR – mobile X-ray systems	165	87.0	0.292	–
		EVAR – hybrid rooms	123	278.0	1.403	–
Regional (Europe)	Tuthill et al.^(^15^)^	Abdominal EVAR	180	158.49	–	18.13

* Reported as the third quartile.

### Variation factors

The variation factors influencing DRL values included equipment technology (such as additional X-ray beam filtration and improved detector sensitivity), the protocols adopted (standard vs low-dose), equipment quality control (accuracy of dose metrics like KAP and CAK), operator experience, patient characteristics, and procedural features. Schegerer et al.^(^20^)^ stated that newer X-ray equipment tends to offer better filtration and detector capabilities. Rial et al.^(^18^)^ observed that procedures performed in hybrid rooms resulted in higher DRL values than did those involving the use of C-arm systems. In additionally, some authors stressed the need for routine quality control tests to ensure the accuracy of dose measurements, particularly regarding correction factors for KAP and CAK.

No association was identified between DRL values and the type of institution, as evidenced by the multicenter study that analyzed potential variations between private clinics and public hospitals^(^16^)^. Operator experience^(^21^,^32^,^37^,^38^)^ was another reported variation factor deemed determinant for patient dose. Less experienced operators, such as interns and residents, tend to take longer to perform procedures, and consequently, the dose is proportionally higher. Peter et al.^(^32^)^ reported that when a procedure is considered more complex/challenging, it is performed by more experienced physicians, resulting in lower radiation doses.

As a dose optimization strategy, dedicated low-dose protocols have been reported. Forbrig et al.^(^23^)^ reported that a dedicated low-dose fluoroscopy protocol resulted in a 33% reduction in radiation exposure. Low-dose protocols in digital subtraction angiography have also been documented^(^26^,^27^,^31^,^35^)^, resulting in dose reductions ranging from 20% to 61%. Changing the exposure mode (from normal to low) and using the pulsed fluoroscopy mode have also been shown to decrease patient radiation doses^(^32^,^37^)^.

### Trend analysis

A trend analysis could assess changes in DRL establishment processes and radiation exposure over time, considering improvements in technology and clinical practice. However, none of the studies reviewed the same procedures at the same centers or evaluated temporal changes in DRL values and optimization strategies. All of the studies presented cross-sectional data, which precluded the evaluation of DRL implementation outcomes within institutions.

### Practical recommendations

Practical recommendations included standardizing procedural nomenclature to improve dose comparisons^(^21^)^, establishing separate DRLs for therapeutic and diagnostic procedures^(^28^)^, and encouraging the use of dose tracking software for data collection and DRL establishment, including complexity considerations^(^36^,^39^)^.

## DISCUSSION

This review provides a comprehensive overview of the scientific evidence available to establish DRL values in interventional radiology. The studies evaluated addressed a wide range of procedures. However, the lack of standardization in nomenclature across studies hindered the direct comparison of DRL values. The results indicated a predominance of studies conducted in Europe, especially in Germany. The complexity of procedures and the lack of consideration of that complexity were recurring challenges faced by researchers, suggesting the need for more refined approaches that include this factor. In addition, the use of dose management software has emerged as an effective strategy to facilitate data collection and the establishment of DRLs. Examples of commonly used dose management systems include DoseWatch (GE Healthcare, Buc, France), Radimetrics (Bayer Healthcare, Whippany, NJ, USA), and OpenREM (an open-source platform: https://openrem.org/).

The breadth of interventional radiology was clearly highlighted with the identification of DRL values for 113 different procedures. Given that the DRL establishment process can be considered a form of optimization, it is recommended that institutions assess their typical dose values or, when available, evaluate local DRL values in relation to national or regional DRL benchmarks^(^5^)^. For this, it is essential that procedures have standardized terminology. Although the World Health Organization provides the International Classification of Health Interventions as an online reference^(^40^)^, it was not employed in any of the studies evaluated in this review.

In the quest to define DRLs, studies coming out of Europe have predominated. In 2013, the European Union published Council Directive 2013/59/Euratom^(^41^)^, which mandated member states to inform patients about the radiation dose received during procedures and to establish DRLs^(^42^)^. In this context, investments were made in dose management systems. This can be observed in this review in terms of practical recommendations from the majority of articles. National or regional regulatory frameworks play a crucial role in strengthening the implementation of the DRL establishment process.

The analysis of procedural complexity should take into account variations in anatomy and clinical factors (e.g., body habitus, vascular anatomical variations, diameter of normal vessels, and number of vessels to be treated) that determine technical parameters and FT^(^5^)^, directly impacting DRL values. That level of structure was not observed among the articles evaluated in this review. The absence of an established standard for defining procedural complexity hinders researcher understanding of how to consistently conduct and compare this analysis^(^37^)^, and all of the studies emphasized the need to include this factor in future research.

This review has some limitations. The predominance of studies conducted in Europe may limit the generalizability of the findings to other regions. Although standardization across diverse procedures was necessary, the considerable heterogeneity in DRL values and methodologies complicated direct comparisons. In addition, despite using comprehensive descriptors, the search strategy may not have captured all relevant interventional radiology procedures.

This systematic review revealed significant gaps in the global implementation of DRLs in interventional radiology. Standardizing procedural nomenclature and complexity is essential for consistent data collection and comparisons. Expanding data collection to underrepresented regions, especially Latin America and, in particular, Brazil, is crucial. European legal frameworks have proven effective in promoting radiation safety, underscoring the need for national regulatory initiatives.

To further enhance the implementation of DRL establishment processes, it is recommended that the adoption of dose tracking and management software be encouraged, that national standardization efforts following the European model be proposed, and that the involvement of centers in Brazil and the rest of Latin America in collaborative data networks be actively promoted. Such coordinated efforts are fundamental to enhancing the effectiveness, reproducibility, and global applicability of DRL initiatives.
